# Glutamine levels in patients with traumatic brain injury and subarachnoid haemorrhage

**DOI:** 10.1186/2197-425X-3-S1-A779

**Published:** 2015-10-01

**Authors:** J van Rosmalen, BS Jakobs, P Vos, HMF Kuijsten, JAH van Oers, D Ramnarain

**Affiliations:** St Elisabeth Hospital, ICU, Tilburg, Netherlands; St Elisabeth Hospital, KCHL, Tilburg, Netherlands

## Introduction

In critical illness, the rapid depletion of glutamine has been associated with increased mortality[1]. This has led to the concept that early glutamine suppletion would benefit these patients.

A recent trial[2] however showed that early suppletion of glutamine was associated with an increased mortality. In a majority of patients glutamine levels were within normal range at admission. Patients with severe traumatic brain injury (TBI) and subarachnoid haemorrhage (SAH) however, were excluded in this trial. In literature no data on glutamine levels are available in critically ill neurologic patients.

## Objectives

We conducted an observational pilot study measuring glutamine levels in the first week after TBI and acute SAH.

## Methods

In a 30-bed intensive care unit of a teaching hospital patients admitted with TBI (n=5) and SAH (n=5) were selected. Plasma glutamine levels were measured at admission and on six consecutive days. Glutamine deficiency was defined as a plasma glutamine level of less than 420 µmol/L. Optimal nutrition per patient was calculated by a dietician. Jevity^®^ standard, Plus and HiCal (Abbott Nutrition) were used containing 0.36-0.40 gram glutamine/100 kcal. Actual intake was noted in a Patient Data Management System (Metavision®, iMDsoft). Data were collected in Excel (Microsoft®) and analysed with SPSS^®^ (IBM).

## Results

See Table 1.

**Table 1:** Table 1

	Glutamine <420 µmol/L	Glutamine >420 µmol/L
N=10	n=7	n=3
Age (median, years)	60	54
Gender (male/female, %)	29/71	67/33
Type of patient (SAH/TBI, %)	43/57	67/33
APACHE II	18.4	19.0
Mechanical ventilation (days)	8.4	9.3
Length Of Stay (days)	15.9	17.0
Hospital-Length Of Stay (days)	22.1	40.7
3-Month mortality (%)	57	0

**Figure 1:**
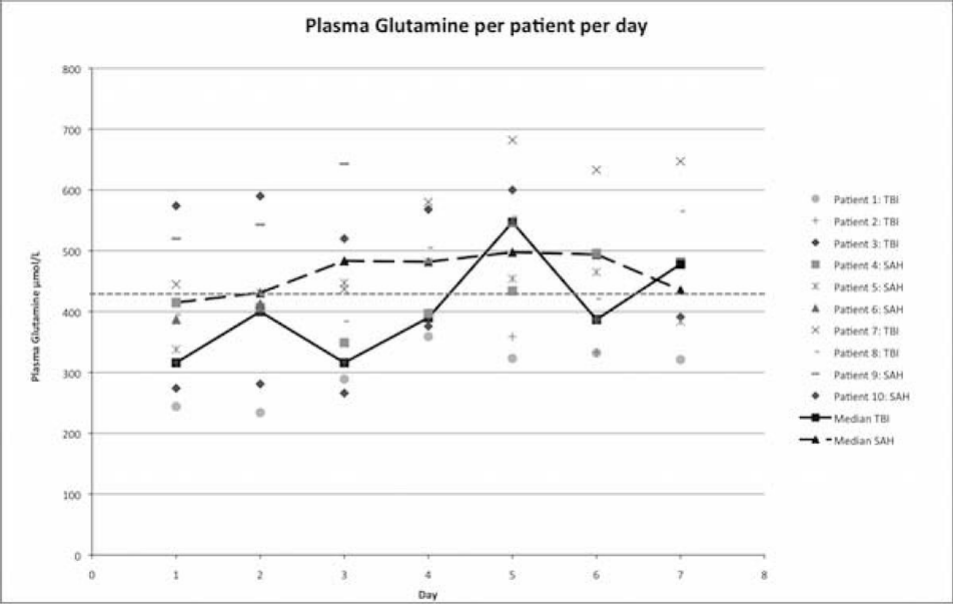
Figure 1

Mean glutamine level in TBI and SAH patients was 334.8 ± 83.7 µmol/L and 446.8 ± 97.4 µmol/L at admission. Glutamine deficiency was common: 7 out of 10 patients were deficient according to the definition (Table 1.) Optimal intake per individual was calculated to be 2022 ± 498 kcal/120 ± 36 grams of protein. Due to several causes i.e. gastric retention and passage disorders, actual intake was 1480 ± 580 kcal and 77 ± 35 grams of protein, primarily via nasogastric tube feeding. This was 72% and 71% of calculated optimal calorie and protein intake. After seven days of treatment and nutritional support glutamine levels increased significantly in all patients with TBI (451 ± 146.5 µmol/L) and SAH (476 ± 84.2 µmol/L). In one SAH patient glutamine level declined but remained in normal range (Figure 1.).

## Conclusions

We found low glutamine levels in 70% of TBI and SAH patients at admission but no evidence of glutamine depletion during treatment. Intake of only 72% of calculated optimal calorie intake and 71% of protein intake was sufficient to reach near-normal levels of glutamine in patients with TBI and SAH.
